# Gold Nanoparticles in Biology and Medicine: Recent Advances and Prospects 

**Published:** 2011

**Authors:** L.A. Dykman, N.G. Khlebtsov

**Affiliations:** Institute of Biochemistry and Physiology of Plants and Microorganisms, Russian Academy of Sciences; Saratov State University

**Keywords:** gold nanoparticles, plasmon resonance, biosensors, biomedical diagnostics, photothermal and photodynamic therapy, targeted drug delivery, nanotoxicology

## Abstract

Functionalized gold nanoparticles with controlled geometrical and optical properties are the subject of intensive studies and biomedical applications, including genomics, biosensorics, immunoassays, clinical chemistry, laser phototherapy of cancer cells and tumors, the targeted delivery of drugs, DNA and antigens, optical bioimaging and the monitoring of cells and tissues with the use of state-of-the-art detection systems. This work will provide an overview of the recent advances and current challenges facing the biomedical application of gold nanoparticles of various sizes, shapes, and structures. The review is focused on the application of gold nanoparticle conjugates in biomedical diagnostics and analytics, photothermal and photodynamic therapies, as a carrier for delivering target molecules, and on the immunological and toxicological properties. Keeping in mind the huge volume and high speed of the data update rate, 2/3 of our reference list (certainly restricted to 250 Refs.) includes publications encompassing the past 5 years.

## CONTENTS 

Introduction 

1. Gold nanoparticles in diagnostics 

1.1. Visualization and bioimaging methods 

1.2. Analytic methods for diagnostics 

1.2.1. Homophase methods 

1.2.2. Dot-immunoassay 

1.2.3. Immune chromatography 

1.2.4. Plasmon resonance biosensors 

2. Gold nanoparticles in therapy 

2.1. Photothermal therapy using gold nanoparticles 

2.2. Photodynamic therapy using gold nanoparticles 

2.3. Use of gold nanoparticles as therapeutic agents 

3. Gold nanoparticles as drug carriers 

4. Immunological properties of gold nanoparticles 

5. Biodistribution and toxicity of gold nanoparticles 

Conclusion 

## INTRODUCTION 

Gold is one of the first metals to have been discovered; the history of its study and application spans at least several thousand years. The first data on colloidal gold can be found in treatises by Chinese, Arabian, and Indian scientists, who managed to obtain colloidal gold as early as in the V–IV centuries BC. They utilized it for medicinal purposes (Chinese “golden solution” and Indian “liquid gold”), amongst other uses. In Europe during the Middle Ages, colloidal gold was studied and used in alchemist laboratories. Paracelsus wrote about the therapeutic properties of gold quintessence — “ *quinta essentia auri,* ” which he obtained via the reduction of gold chloride by vegetable extracts in alcohols or oils. He used the “potable gold” for the treatment of a number of mental diseases and syphilis. His contemporary, Giovanni Andrea, used “ *aurum potabile* ” as a therapy for patients with leprosy, plague, epilepsy, and diarrhea. In 1583, the alchemist David de Planis-Campy, who served as doctor to Louis XIII of France, recommended his “longevity elixir,” a colloidal solution of gold in water. The first book on colloidal gold preserved to our days was published in 1618 by the philosopher and doctor of medicine Francisco Antonii [1]. It contains data on how to obtain colloidal gold and its application in medicine, including practical advice.

**Fig. 1:**
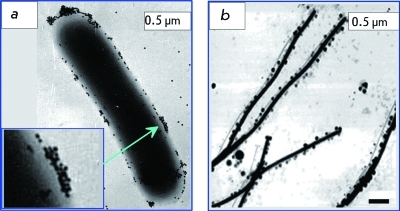
TEM image of a *Listeria monocytogenes* cell (a) and AFM image of a tobacco mosaic virus labeled by colloidal gold nanoparticles conjugated with the corresponding antibodies. (b). Adapted from Refs. [11] (а) and [12] (b).

Despite its centuries-old history, the “revolution in immunochemistry” associated with the use of gold nanoparticles (GNP) in biological studies occurred only in 1971, when the British researchers Faulk and Taylor [2] described a method of antibody conjugation with colloidal gold for direct electron microscopy visualization of the surface antigens of salmonellae. The study was initiated using biospecific markers – colloidal gold conjugated with immunoglobulins and other molecules – in different spheres of biology and medicine. Over the past 40 years, there have been many studies devoted to the application of functionalized nanoparticles – conjugates with recognizing biomacromolecules (antibodies, lectins, enzymes, aptamers, etc.) – in biochemistry, microbiology, immunology, cytology, plant physiology, morphology, etc.

The range of GNP use in modern medical and biology studies is extremely wide. In particular, it comprises genomics, biosensorics, immunoanalysis, clinical chemistry, detection and photothermolysis of microorganisms and cancer cells; the targeted delivery of drugs, DNA and antigens; optical bioimaging and the monitoring of cells and tissues using modern registration systems. It has been argued that gold nanoparticles could be used in almost all medical applications: diagnostics, therapy, prevention, and hygiene. A wealth of information on how to obtain and use colloidal gold in biology and medicine, as well as how it functions, can be found in books and reviews [3–8]. The broad range of applications for GNP is based on their unique physical and chemical properties. In particular, the optical properties of GNP are determined by their plasmon resonance, which is associated with the collective excitation of conduction electrons and localized in the broad region, from the visible to the infrared (IR) region, depending on the particle size, shape, and structure [9].

Taking into account the large volume of data published and the high speed at which they are updated, our review aimed to generalize the results obtained over the past several years in the most promising directions in the use of GNP in modern medical and biological studies. 

## 1. GOLD NANOPARTICLES IN DIAGNOSTICS 

**1.1. Visualization and bioimaging**

Gold nanoparticles have been in active use in the identification of chemical and biological agents. Electron microscopy (predominantly, transmission electron microscopy — TEM) has historically remained the predominant means to detect biospecific interactions using colloidal gold particles (due to their high electron density). It is not by happenstance that the first three-volume publication about the application of colloidal gold [10] was chiefly devoted to TEM using GMP. The use of high-resolution instruments (high-resolution transmission electron microscope – HRTEM) and systems of digital recording and the processing of images are examples of the modern application of electron microscopy equipment. The main practical use of immune electron spectroscopy in modern medico-biological studies is for the identification of causative agents of infectious diseases and their surface antigens [11] ( *Fig. 1A* ). Scanning probe microscopy [12] ( *Fig. 1B* ), scanning electron microscopy [13], and fluorescence microscopy [14] are frequently used for the same purpose.

**Fig. 2:**
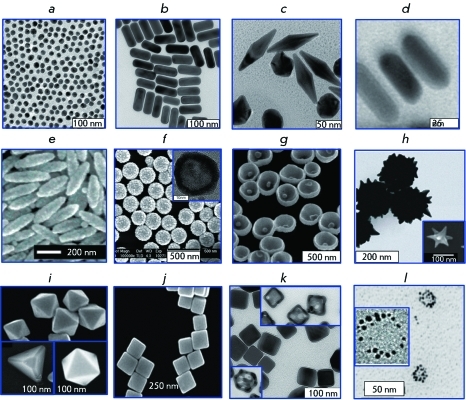
Various types of gold plasmon-resonance nanoparticles: 16-nm nanospheres (a) [8], nanorods (b) [15], bipyramids (с) [16], gold nanorods with silver coating (d) [17], “nanorice” – Fe _2_ O _3_ nanorods covered by a gold nanoshell (e) [18], gold nanoshells onto silica cores, SiO _2_ /Au (f) [19] (the inset shows a hollow gold nanoshell [20]), nanobowls with a gold seed on the bottom (g) [21],“spiky nanoshells” with SiO _2_ /Au cores [22] (the inset shows a “nanostar” particle [23]) (h), tetrahedra, octahedra, and cubooctahedra (i) [24], nanocubes (j) [24], silver nanocubes and gold-silver nanocages obtained with silver cube templates (insets) (k) [25], nanonecklaces [26] (l). The figures were adapted from the cited works. The figures are reproduced by permission from The Royal Society of Chemistry (http://dx.doi.org/10.1039/b711490g, http://dx.doi.org/10.1039/c0cs00018c), The PCCP Owner Societies (http://dx.doi.org/10.1039/b925102b) and The American Chemical Society.

In addition to the conventional colloidal gold with quasi-spherical particles (nanospheres), non-spherical particles, such as nanorods, nanoshells, nanocages, nanostars, and other types of particles (this group of particle were named “plasmon resonance particles of noble metals”) have recently been used [8] ( *Fig. 2* ).

The visualization methods with the use of GNP and optical microscopy [27], in particular, confocal laser microscopy, have gained increasing popularity in medical and biological research. Confocal microscopy is a method for the detection of micro-objects using an optical system, which permits the registering of light radiation only from the objects located in its focal plane; therefore, the scanning of samples along their height can be performed, and their 3D images can be obtained by superposition of scanograms. The use of GNP and antibody–GNP conjugates allows for real-time detection of the penetration of gold into living cells (e.g., cancer cells) at the level of a single particle and even for the estimation of their amount [28].

**Fig. 3:**
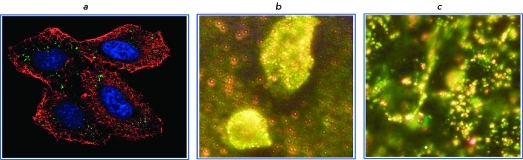
a - Confocal image of HeLa cells in the presence of gold nanoparticles. The nucleus was stained with a Hoechst 33258 reagent (in blue), whereas the actin cytosceleton was stained with an Alexa Fluor 488 labeled falloidine (in red), and gold nanoparticles (in green) were detected by two-photon luminescence [30]. A dark-field microscopic image of cancer (b) and healthy (c) cells with gold nanoparticles conjugated with antibodies to EGRF [31]. Adapted from the cited works by permission from The American Chemical Society.

The methods for obtaining confocal images include fluorescence detection (confocal fluorescence microscopy) or resonance elastic or two-photon (multiphoton) light scattering by plasmon nanoparticles (resonance scattering confocal microscopy or two-photon luminescence confocal microscopy). These techniques are based on detecting micro-objects using an optical microscope in which the object’s luminescence is excited due to the simultaneous absorption of two (or more) photons; the energy of each of them being lower than that required for fluorescence excitation. The major advantage of this method is that the strong decrease in the background signal results in the contrast being enhanced. The use of two-photon luminescence of gold nanoparticles allows to visualize (amongst other objects) oncomarkers on the surface or inside a cell [29, 30]. *Fig. 3A* provides an example of combined bioimaging of a malignant cell using adsorption, fluorescence, and luminescence plasmon resonance labels.

Dark-field microscopy based on light scattering by microscopic objects (resonance scattering dark-field microscopy), including objects with a size less than the resolution limit of a light microscope, remains one of the most popular methods in bio-imaging using GNP ( *Figs. 3B, C* ). Upon dark-field microscopy, only the light scattered by an object under lateral illumination can reach the lens (similar to the Tyndall effect); therefore, the scattering object shines brightly against the dark background. Gold nanoparticles offer more possibilities for the detection of biospecific interactions using dark-field spectroscopy in comparison with fluorescence labels [8], since the scattering cross-section of a particle is higher than the fluorescence cross-section of one molecule by 3–5 orders of magnitude. This principle was applied by American researchers at the El-Sayed laboratory [31] in a new method for a simple and reliable diagnostics of oncologic diseases with the use of GNP. The method is based on the preferential binding of GNPs conjugated with antibodies specific to tumor antigens to the surface of cancerous cells, as compared with binding to healthy cells. Thus, resonance scattering dark-field microscopy can be used to map a tumor with an accuracy of up to several cells ( *Figs. 3B, C* ). In subsequent studies, gold nanorods [32], nanoshells [33], nanostars [34], and nanocages [35] were used with the same purpose.

Nanocages belong to a relatively new family of nanoparticles fabricated by galvanic replacement on silver nanocube templates. In this reaction, three silver atoms are replaced by a single gold atom, resulting in the gradual formation of various porous alloy structures of gold and silver, which are called nanoboxes and nanocages [35]. In the formation process of these particles, the plasmon resonance shifts from 430–440 nm for cubes to 700–900 nm for nanocages.

The use of nonspherical and/or heterogeneous particles, as well as self-assembling particle monolayers or island films, opens up new opportunities to enhance sensitivity in detecting biomolecular binding on or near the surface of nanostructures. The principle of amplification of the biomolecular binding signal is based on inducing strong local electromagnetic fields near particles with sharp regions on their surface or in the narrow (on the order of nanometer or less) gaps between two nanoparticles. It stipulates enhanced sensitivity of plasmon resonance to the local dielectric environment and a high scattering intensity in comparison with spheres of the same volume. Therefore, these nanostructures can be considered as having significant potential for application for biomedical diagnostics purposes using dark-field microscopy [36].

Gold nanoparticles are used in resonance scattering dark-field microscopy for the detection of microbial cells and their metabolites [37], the bio-imaging of tumor cells [38], and for the detection of receptors on their surface [39], and for the study of endocytosis [40]. In most biomedical applications, the efficacy of labelling cells with conjugates is assessed at the qualitative level. The method of quantitative assessment of the efficacy of cell labelling with gold nanoparticles that was used for labelling pig embryo kidney cells with gold nanoshell conjugates is one of the few exceptions [41].

In addition to the aforementioned methods used to detect biospecific interactions using different variants of optical microscopy and GNP, other modern methods for detecting and bio-imaging have recently been in active development; these methods can be combined under the general name “biophotonic methods” [9]. Biophotonics combines all studies associated with the interaction between light and biological cells and tissues. Biophotonic methods include optical coherence tomography [42], X-ray and magneto-resonance tomography [43, 44], photoacoustic microscopy [45] and tomography [46], fluorescence correlation microscopy [47], etc. Gold nanoparticles of various sizes and shapes are also successfully used in these methods. We believe that biophotonic methods with the use of gold non-spherical nanoparticles may prove to be of considerable promise for *in vivo* bioimaging [48]. Moreover, the significance of a new class of GNP conjugates with recognized constructions based on the barnase–barstar module [49] should be noted.

**1.2. Analytic methods for diagnostics**

**Fig. 4:**
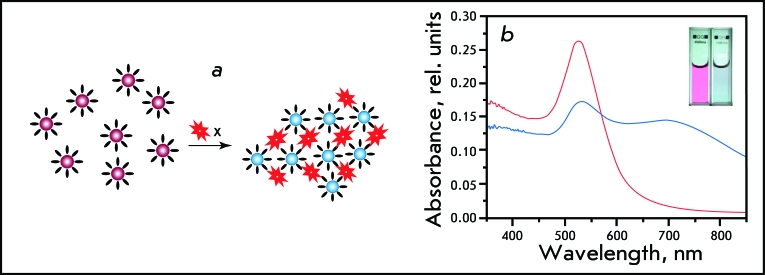
Sol-particle immunoassay: a scheme of conjugate aggregation caused by binding to target molecules (a) and corresponding changes in the sol color and absorption spectra (b). Adapted from Ref. [52] by permission from The American Chemical Society.

*1.2.1. Homophase methods.* Beginning in the 1980s, conjugates of colloidal gold and recognizing biomacromolecules began to be used in various analytic methods of clinical diagnostics. In 1980, J. Leuvering *et al* . [50] proposed a new method that was called sol particle immunoassay (SPIA). This method is based on two principles: 1) the color and absorption spectrum of a sol vary little upon biopolymer adsorption on individual particles [51]; 2) when particles approach a distance that is less than one-tenth of their diameter, the sol’s red color changes into purpuric; the absorption spectrum broadens and shifts into the red region [51]. These changes in the absorption spectrum can be easily detected either spectrophotometrically or visually ( *Fig. 4* , [52]).

An optimized version of this method (using larger gold particles and monoclonal antibodies to various sites of an antigen) was applied to detect chorionic gonadotropin in the urine of pregnant women [53]. On the basis of these elaborations, Chefaro Company (Netherlands) launched the Discretest™ kit for early out-of-hospital diagnosis of pregnancy. Kits for immune colorimetric determination of the rheumatoid factor and streptolysin are produced by PLIVA Lachema Company (Czech Republic).

This method was subsequently used for performing immunoassay of the antigens of schistosomes and rubella viruses and for the quantitative determination of immunoglobulins (refs. in [5]), for determining thrombin (using aptamers) [54] and glucose [55], for the direct detection of cancer cells [56] and leptospira cells in urine [57], and for determining markers of Alzheimer’s disease [58] and protease activity [59]. The simultaneous use of conjugates of gold nanorods and nanospheres with antibodies for detecting tumor antigens was described in [60]. The data on the determination of the hepatitis B virus in blood using gold nanorods conjugated with specific antibodies were published in [61].

The implementation of all versions of the SPA method proved to be relatively simple but at the same time both highly sensitive and specific. However, in a number of cases, despite the evident complementarity of a pair, no aggregation took place; the solution’s color and the absorption spectra either did not change or changed to an insignificant degree. The model of formation of the second protein layer on gold particles without a loss in the aggregate stability of the sol was suggested in [62]. The changes in spectra caused by adsorption of biopolymers on the surface of gold nanoparticles are relatively small. However, even such negligible changes in absorption spectra resulting from the change in the biopolymer layer structure (or, equivalently, in its average refractive index) near the GNP surface can be recorded and used for a quantitative analysis in biological applications [63].

Various optical methods, including different versions of IR Fourier [64] and UV-vis beam absorption or deflection spectroscopy (see refs. in [5]), hyper-Rayleigh [65], differential static [51], and dynamic [60] light scattering, as well as surface-enhanced Raman scattering [66], have been used to enhance the sensitivity of the analytical homophase reaction.

A new version of the SPIA method was proposed by C. Mirkin  *et al* . [67] for the colorimetric detection of DNA. Currently, the colorimetric determination of DNA involves two strategies: (1) the use of GNP conjugated with thiol-modified single-stranded DNA [67–71] and (2) the use of nonmodified GNP [72, 73]. The first strategy is based on the aggregation of conjugates of 10–30 nm GNP with thiol-modified single-stranded DNA probes upon introduction of target polynucleotides into the system. In this case, probes of two types are used, which are complementary to two terminal regions of the targets. Hybridization of targets and probes results in the formation of GNP aggregates, which is accompanied by changes in the absorption spectrum of the solution and can be easily detected visually, photometrically [74], or via dynamic-light scattering [71]. Within the framework of the first strategy proposed by Maeda *et al* . [75], the diagnostic system based on the aggregation of GNP modified by probes of one type upon introduction of DNA targets into the solution under conditions of high ionic strength was used. Meanwhile, Baptista  *et al.* [70] elaborated a method based on the enhanced stability of conjugates upon the introduction of complementary targets even under conditions of high ionic strength (2 M NaCl), and the aggregation of noncomplementary targets was observed. The apparent contradictions between the two approaches were ascribed [76] to the difference in the surface functionalization density.

The second strategy [72] is based on the fact that the single-stranded DNA protects unmodified GNP against aggregation upon high ionic strength, while the formation of duplexes upon hybridization cannot stabilize the system. This approach was used to determine the hepatitis C virus [77]. Xia  *et al.* [78] recently described a new variant of the second strategy in which single-stranded DNA, unmodified GNP, and cationic polyelectrolyte are used. The same approach turned out to be suitable for determining a wide range of targets, including peptides, amino acids, pesticides, antibiotics, and heavy metals. Contrary to the procedures with usual GNP, He  *et al.* [73] proposed a method for determining HIV-1 U5 viral DNA using nanorods stabilized by cetyltrimethylammonium bromide (CTAB) and the light scattering method with a detection limit of about 100 pM. In the optimized version where absorption spectroscopy is used [79], the detection limit was reduced to 0.1 pM. It has been recently demonstrated that positively charged GNP coated with CTAB can be used for the detection of DNA targets in combination with spectroscopy and dynamic scattering methods [15].

**Table 1:** The sensitivity limits of the immunodot/blot methods on nitrocellulose filters using various labels (according to [89])

Label	Sensitivity limit, pg of protein/fraction
^125^I	5
Horseradish peroxidase	10
Alkaline phosphatase	1
Colloidal gold	1
Colloidal gold + silver	0.1
Fluorescein isothiocyanate (FITC)	1000

The enumerated versions of the method of sol particle aggregation due to the hybridization reaction were used to determine the DNA of micobacteria [70], staphylococci [80], streptococci [81], and chlamydiae [82] in clinical samples.

The ability of gold particles to aggregate upon interaction with proteins inducing colour change in the solution served as the basis for the quantitative method of colorimetric determination of proteins [83]. A new version of the SPIA method using microtitration plates, an ELISA reader, and colloidal gold-trypsin conjugates was proposed for the detection of proteins [84].

*1.2.2. Dot immunoassay.* At the early stages of the development of immunoassays, preference was given to liquid phase techniques, in which the bound antibodies were deposited or the unbound antigen was removed using dextran-coated activated coal. The solid-phase techniques have recently been the most widely used (first used in radioimmunoassay of proteins), since they provide the possibility to considerably simplify the analysis procedure and reduce the background signal. The most widespread solid-phase carriers are polystyrene plates and nitrocellulose membranes.

Radioactive isotopes ( ^125^ I, ^14^ C, ^3^ H) and enzymes (peroxidase, alkaline phosphatase, etc.) are widely used as a label in membrane tests (dot and blot analyses). In 1984, four studies were independently published [85–88] in which colloidal gold was used as a label for solid-phase immunoanalysis. The use of GNP conjugates in solid-phase analysis is based on the fact that the intense red coloration of a gold-containing marker allows one to determine visually the results of a reaction that was carried out on a solid carrier. “Immuno-gold techniques” in dot blot assay are superior to the other types of assays (e.g., immunoenzyme assay) in sensitivity ( *Table 1* , [89]), simplicity, speed, and cost. The GNP size after the corresponding immunochemical reaction can be increased using the reaction of amplification with silver [90] or gold salts (autometallography) [91], which considerably broadens the limits of application of this method. The optimized version of solid-phase assay using the Quantity One densitometry system (Bio-Rad, USA) provided a linear range of detection from 1 pM to 1 µM [92] with a limit of 100 aM and its decrease to 100 zM by silver amplification. It should be kept in mind that this record decrease in the detection limit due to silver amplification was attained using the sensitive densitometry system (Quantity One). The modern instrument methods, such as photothermal deflection of a probe laser beam induced by heating of the local environment near the absorbing particles by heating laser pulses (LISNA [93]), also ensure a very broad detection range: from several orders of magnitude to several isolated particles per blot.

When carrying out specific staining, the membrane with the material applied on it is incubated in a solution containing antibodies (or other biospecific probes) labelled with colloidal gold [94]. Immunoglobulins, Fab- and scFv- antibody fragments, protein A, lectins, enzymes, avidin or anti-biotin antibodies upon the study of biotin conjugated samples, aptamers, and other recognizing molecules are used as probes when carrying out “gold” dot or blot assay. Several labels can be simultaneously used as well (e.g., colloidal gold and peroxidase or alkaline phosphatase) to reveal different antigens on a membrane.

Colloidal gold in membrane tests was used to diagnose parasitic, virus, and fungus diseases; tuberculosis, melioidosis, syphilis, brucellosis, shigellosis, and coli-infections; to determine blood groups and pregnancy at an early stage, for dot blot hybridization, and for revealing the diphtheritic toxin, diagnostics of myocardial infarction, and hepatitis B (see refs. in [5]).

Immunodot assay is one of the simplest methods for determining the antigens immobilized on membranes; in some cases, this method allows one to estimate their quantitative content. Most frequently, immunodot assay is used to study soluble antigens [95]. However, few studies have been published in which corpuscular antigens (whole bacterial cells) were studied by dot assay with enzyme labels [96]. The procedure of dot assay in whole bacterial cells with visualization of the reaction products using colloidal gold conjugates as biospecific markers (“cell-gold immunoblotting”) was first used for serotyping soil nitrogen-fixating microorganisms of the *Azospirillum* genus [97]. This method was subsequently used for express diagnostics of enteric infections [98].

**Fig. 5:**
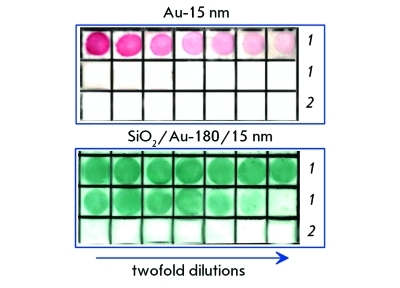
Dot immunoassays of a normal rabbit serum (1) by using 15-nm GNPs and silica/gold nanoshells (180-nm-core diameter and 15-nm gold shell) conjugated to sheep’s antirabbit antibodies. The IgG quantity equals 1 μg for the first (upper left) square and is decreased by twofold dilution (left to right). The bottom rows (2) correspond to a negative control (10 μg BSA in each square). Adapted from Ref. [100].

The results of applying gold nanoshells as biospecific labels for dot assay were first presented in paper [99], where three types of gold nanoshells with diameters of the silicate nucleus of 100, 140, and 180 nm and gold shell thickness of approximately 15 nm were studied. Normal rabbit serum (target molecules) and sheep anti-rabbit immunoglobulins (recognizing molecules) were used as a biospecific pair. When using the standard protocol of the dot assay on a nitrocellulose membrane with 15-nm colloidal gold nanoparticles as labels, the minimum detectable amount of rabbit IgG was equal to 15 ng. The replacement of the colloidal gold conjugates for nanoshells enhanced the assay sensitivity to 0.2 ng in the case of gold nanoshells of 180/15 nm, and to 0.4 ng in the case of gold nanoparticles of 100/15 and 140/15 nm type ( *Fig. 5* ). Such a noticeable increase in the sensitivity of the dot assay with nanoshells, in comparison with colloidal gold, was explained by the differing optical properties of the particles [100].

The use of GNP seems to have a high potential for analyzing large arrays of antigens in micromatrices (immunochips) [101], which permits the simultaneous determination of the analyzed compound in 384 samples at a concentration of 60–70 ng/l or (taking into account the microliter amounts of the sample and the immunogold marker) with a detection limit of less than 1 pg.

*1.2.3. Immunochromatography.* Approximately 10 years ago, several foreign companies launched immunochromatographic test systems for instrument-free diagnostics. Due to the high specificity and sensitivity of the immunoassay, these tests found wide application in determining narcotic agents, toxins, early diagnostics of pregnancy, and screening of extremely dangerous and urogenital infections. New methods for the diagnostics of tuberculosis, helicobacteriosis, staphylococcus infection, hepatitis B, prostatitis, determining pregnancy at the early stages, pesticides, aflatoxin, diethylstilbestrol and cephalexin in the environment, and DNA hybridization have been elaborated (see refs. in [5]).

Immunochromatographic assay [102] is based on eluent motion along the membrane (lateral diffusion), resulting in the formation of specific immune complexes that are detected as stained bands on different membrane regions. Enzymes, stained latexes, and quantum dots [102] are used as labels in these systems; however, in the overwhelming majority of cases, gold nanoparticles are used [103].

The sample under investigation migrates along the test strip due to capillary forces. If a sample contains the desired compound or immunologically close ones when the sample passes through the absorbing device, a reaction with specific antibodies labelled with colloidal gold occurs, accompanied by the formation of an antigen–antibody complex. The colloidal preparation is involved in the reaction of competitive binding with the antigen immobilized in the test zone (haptene conjugated with a protein carrier is usually used for immobilization in the detection of low-molecular-weight compounds). If the concentration of antigens in the sample is higher than the threshold level, the conjugate has no vacant valences for interacting in the test zone; the stained band corresponding to complex formation is not observed. If the sample does not contain the desired compound, or its concentration is lower than the threshold level, antigen immobilized in the test zone of the strip reacts with antibodies on the surface of colloidal gold, which results in the formation of a stained band. 

**Fig. 6:**
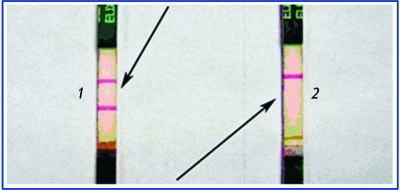
Positive (1) and negative (2) results of an immunochromatographyc assay.

As the liquid front moves further, gold particles with immobilized antibodies, which did not react with the antigen in the test zone of the strip, are bound to antispecific antibodies in the control zone of the test strip. The emergence of a stained band in the control zone attests to the validity of the testing procedure and the diagnostic activity of the components of the system. The negative testing result, the emergence of two stained bands (in the test zone and the control zone), points to the fact that the sample contains no antigen or its concentration is lower in comparison with the threshold level. The positive testing result, the emergence of a single stained band in the control zone attests to the fact that antigen concentration is higher than the threshold concentration ( *Fig. 6* ).

**Fig. 7:**
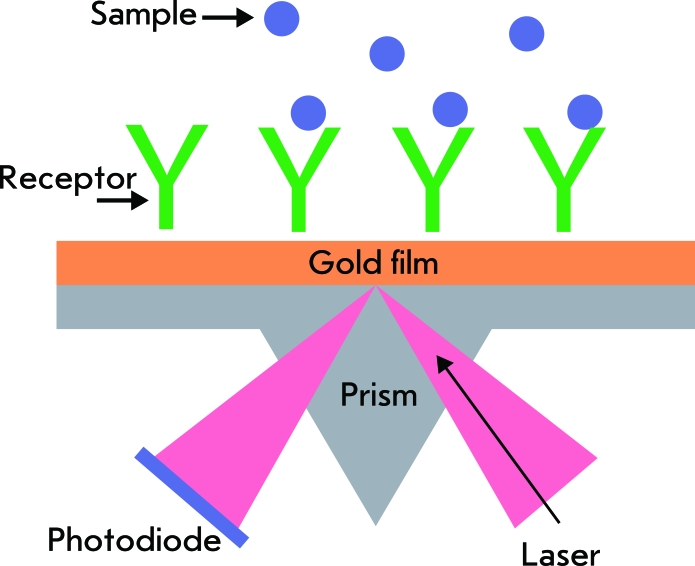
Scheme for detection of target molecules with a BIAcore™ device based on a total internal reflection prism covered by a thin gold layer. Adapted from Ref. [106].

The study of these test systems has demonstrated their high stability, the reproducibility of the results, and correlation with alternative methods. Densitometric characterization of the degree of heterogeneity of the samples stands at 5–8%, which enables one to visually assess the results of the analysis with an appreciably reliable accuracy. These tests are very simple and convenient to use. 

*1.2.4. Plasmon resonance biosensors.* Over the past years, gold and silver nanoparticles and their composites have found broad application as efficient optical detectors of biospecific interactions [104]. In particular, the resonance optical properties of nanometer-sized metal particles have found successful application in the design of so-called biochips and biosensors. There are many types of sensors, *viz.* colorimetric, refractometric, electrochemical, piezoelectric, and certain others [102, 105, 106]. These devices are of great interest in biology (determination of nucleic acids, proteins, and metabolites); medicine (drug screening, analysis of antibodies and antigens, diagnostics of infectious diseases); and chemistry (environmental express monitoring, quantitative analysis of solutions and dispersed systems).

The study of the biospecific interactions in such systems, where GNP are represented by ordered structures (self-assembling thin films) [107] or within polymer matrices [108], has been developing for over a decade. In this case, the amplification of the optical signal from the conjugate due to the strengthening of the exciting local field in an aggregate that was formed from gold nanoclusters is used. New unique technologies are currently being used for the design of biosensor devices, including monolayer self-assembly of metal particles (see [109] and refs therein), nanolithography [110], vacuum evaporation [111], etc. It is of fundamental significance to note that particle size and shape [112], interparticle distance [113], and the optical properties of the local environment [114] have a considerable effect on the optical response obtained from nanoparticles or their aggregates (in particular, the ordered ones), which provides the possibility of controlling the sensor’s “tuning.” These properties of metal clusters served as the basis for the design of new promising plasmon resonance biosensor systems (SPR-biosensors) based on the conversion of biospecific interactions into an optical signal. The theory behind the designing process and variants of practical application of such systems were considered in reviews [115–119].

Sensor sensitivity, stability, and selectivity directly depend on the characteristics of the optical registration system. BIAcore™ is the most popular sensor system of this kind [120]. The measurement principle in planar, prismatic, or mirror biosensors is analogous to the principle used in the method of frustrated total internal reflection, which has been conventionally used to measure the thickness and the refractive index of ultrathin organic films on metal (reflecting) surfaces [105]. Plasmon resonance excitation in a planar gold layer occurs when polarized light falls onto the surface at a certain angle. The electromagnetic fields running along the boundary of the surface and localized in its proximity due to the exponential decrease in the amplitude perpendicular to the dielectric with a typical attenuation distance of up to 200 nm are excited on the metal/dielectric interface (the effect of total internal reflection, *Fig. 7* ). The index of refraction at a certain angle and at a certain wavelength depends on the dielectric properties of the thin layer on the interface, which are determined in the final analysis by the concentration of target molecules in the layer.

Various types of biosensors using GNP have been developed for immunodiagnostics of tick-borne encephalitis [121], human papilloma [122] and immunodeficiency-associated [123] viruses, Alzheimer’s disease [124], the determination of phosphororganic compounds and pesticides [125], antibiotics [126], allergens [127], cytokines [128], hydrocarbons [129], immunoglobulins [130], for detecting tumor [131] and bacterial [132] cells, and for determining the activity of cerebral cells [133].

**Fig. 8:**
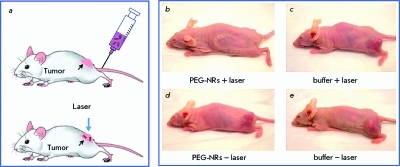
Scheme (left) and photothermal therapy of tumor-burdened mice (2-3 weeks after injection of MDA-MB-435 human cancer cells into opposite flanks). Laser irradiation (a, b, 810 nm, 2 Wt/cm ^2^ , 5 min) were performed 72 h after injection of PEG-coated gold nanorods (NR) (a, с, 20 mg Au/kg) or saline buffer (b, d). It can be seen that the irradiation without particles (control b), as well as the injection of nanorods or saline without irradiation (controls c and d), had no destructive effect, whereas the nanoparticle and laser treatment completely destroyed tumor. Adapted from Ref. [145] by permission of the Publisher.

GNP-based biosensors are applied not only in immunoanalysis [134], but also for the detection of nucleotide sequences [67, 119]. A record sensitivity was achieved for these sensors in pioneer studies [135, 136] in the zepto-molar range based on the recording spectra of resonant scattering from individual particles. This opened the door for the registration of intermolecular interactions at the level of individual molecules [137].

## 2. GOLD NANOPARTICLES IN THERAPY 

**2.1. Photothermal therapy using gold nanoparticles**

Photothermal cell damage is a promising direction in both tumor therapy [138] and the therapy of infectious diseases, which has been intensively developing. The essence of this technique is as follows: gold nanoparticles reach their absorption maximum in the visible or near-infrared region and become hot when irradiated at the corresponding light wavelength. If they are located inside or around the target cells (which can be achieved by conjugation of gold particles with antibodies or other molecules), these cells die.

Thermal exposure has been used in tumor therapy since the 18th century. To do that, both local heating (using microwave, ultrasound, and radio radiation) and hyperthermia of the entire organism [139] (heating to 41–47°С for 1 h) [139] were applied. Upon local heating to 70°С, the duration of the procedure can be reduced to 3–4 min. Local and general hyperthermia result in irreversible cell damage caused by the disruption of the cell’s membrane permeability and protein denaturation. Healthy tissues are also clearly damaged in this process. All this imposes considerable restrictions on the application of this method.

The revolution in cancer thermotherapy was triggered by the use of laser radiation, which made controlled and directed damaging of tumor tissues possible [140]. The combination of laser radiation with fiber-optic waveguides gave excellent results and was named interstitial laser hyperthermia [141]. The disadvantages of laser therapy include the low selectivity associated with the necessity of using powerful lasers for the efficient stimulation of tumor cell death.

In 2003, GNP were applied for the first time as agents for photothermal therapy [142, 143]; it was latter proposed to refer to this kind of therapy as plasmonic photothermal therapy (PPTT) [139]. A new method for selective damaging of target cells, which is based on the use of 20–30 nm gold nanospheres radiated by 20 ns laser pulses (532 nm) in order to create local warming-up, was described in [144]. The sandwich technology consisting in labeling T-lymphocytes with GNP conjugates was used for the pulse photothermy in the model experiment. The use of GNP for the photothermal therapy of chemotherapy-resistant types of cancers seems to be the most promising direction. As opposed to photosensitizers (see below), GNP appear unique because the cells retain their optical properties under certain conditions for a significant amount of time. Successive irradiations with several laser pulses allows to control cell inactivation using a method that is not traumatic, while the use of the nanoparticles, properties to simultaneously scatter and absorb radiation makes PPTT possible using optical tomography [33].

*Fig. 8* represents an example of the successful therapy of induced tumors in mice [145]. Further development of PPTT and its introduction in clinical practice will depend on how successful scientists will be in solving a host of problems, the most significant ones being 1) selecting nanoparticles with the optimal optic properties; 2) increasing the contrast of nanoparticle accumulation in a tumor and decreasing overall potential toxicity; and 3) elaborating methods for delivering optical radiation to the targets and searching for alternative irradiation sources, which would combine high permeation ability with the possibility of GNP heating.

The first requirement is determined by the coincidence of the spectral position of the maximum of the plasmon absorption resonance and the biotissue transparency window in the near-infrared region (700–900 nm). The summarizing theoretical analysis of the photothermal efficiency of GNP depending on their size, shape, structure, and degree of aggregation has been published [113]. It was shown that although gold nanospheres are inefficient in the near-infrared range, their aggregates can be very efficient at appreciably small interatomic distances (below 10% of their diameter). Such clusters form both on a cell’s surface and inside cells [146]. Data on the amplification of PPTT due to clusterization were obtained [147, 148]. In particular, it was ascertained [147] that small aggregates consisting of 30 nm particles enable the destruction of cancer cells at an intensity lower than that in the particle-free control by a factor of 20.

The parameters of gold nanoshells and nanorods that are optimal for PPTT were determined [113, 149]. Today, a number of studies have been published in which the application of gold nanorods [32, 150], nanoshells [142, 151], and a relatively new class of particles – gold-silver nanocages [152, 153] – for PPTT is described. The results of a comparison of the efficiency of heating nanorods, nanoshells, and nanocages are provided in [25, 154].

Three fundamental things should be kept in mind in connection with the optimization of the parameters of a particle. First, intrinsic absorption is not the only parameter determining the efficiency of PPTT [155]. The rapid heating of nanoparticles or clusters results in the formation of vapor bubbles [156], which can cause cavitation cell damage upon irradiation with visible [148] or near-infrared light [157]. The efficiency in the formation of vapor bubbles considerably improves upon the formation of nanoparticle clusters [143, 146]. It is possible that it is this effect, instead of the enhanced absorption, that determines the larger extent of cell damage, other conditions being equal [155]. Finally, irradiation of nanoparticles by high-intensity resonance nanosecond IR pulses may result in the destruction of particles as early as after the first pulse (e.g., see [158, 159] and refs. therein). In a series of studies, Lapotko *et al* . (see [160] and refs. therein) focussed their attention on the fact that the heating of GNP and their destruction may result in an abrupt decrease in the photothermal efficacy of “cold” particles tuned to the laser wavelength. The use of femtosecond pulses does not solve this problem because of the low energy supplied; therefore, it is necessary to accurately control the retention of nanoparticles’ properties for the selected irradiation mode.

We shall now turn our attention to the second issue connected with the problem of targeted delivery of nanoparticles into the tumor. This issue has two significant aspects: increasing the contrast in the desired biotarget and decreasing the side effects conditioned by the accumulation of GNP in other organs, primarily in the liver and spleen (see below). Two delivery strategies are typically used. The first strategy is based on GNP conjugation with PEG, and the second one is based on GNP conjugation with antibodies to certain marker proteins of tumor cells. PEG is used to enhance the bioavailability and stability of nanoparticles, resulting in the increase in time of their circulation in blood flow. Citrate-coated gold nanospheres and CTAB-coated nanorods and nanoshells are characterized by low stability in buffer saline solutions. Upon conjugation of nanoparticles with PEG, their stability increases considerably, preventing salt-induced aggregation. 

**Fig. 9:**
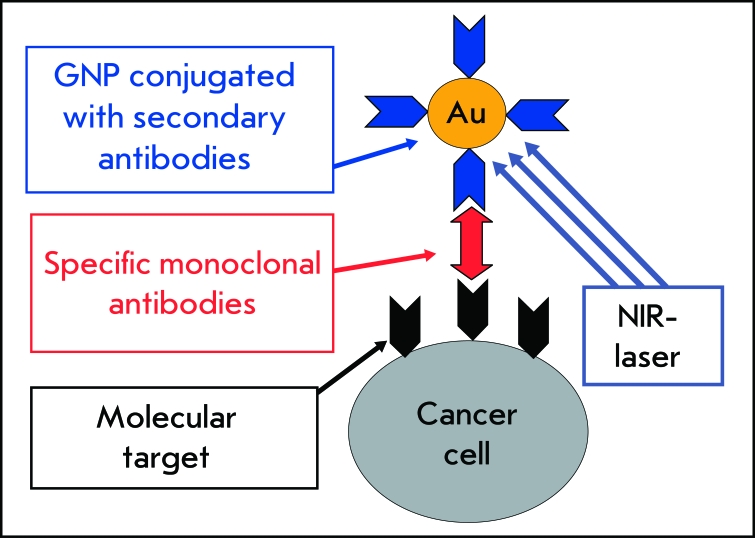
Scheme of the plasmon photothermal therapy with an active delivery of GNPs to cancer cells. Reproduced from Ref. [8] by permission of Elsevier.

PEGylated nanoparticles are preferentially accumulated *in vivo* due to the enhanced permeability of tumor vessels [161] and are retained in it due to the reduced lymphatic drainage. Moreover, PEGylated nanoparticles possess lower availability for the immune system (stealth technologies). This delivery method is called passive delivery, as opposed to the active method, in which antibodies are used [162] ( *Fig. 9* ). The active delivery method is more reliable and efficient. Antibodies to tumor markers are used in it. Most frequently, the epidermal growth factor receptor (EGFR) and its varieties (e.g., Her2) [152, 163], and the tumor necrosis factor, (TNF) [164] serve as such markers. The use of GNP conjugated with antibodies simultaneously for diagnostics and photothermal therapy (the so-called theranostics methods) seems to be the most promising [165]. In addition to antibodies, folic acid, ligand of numerous folate receptors of tumor cells [150], and hormones [166] can be used for active delivery.

The question of the efficacy of targeted delivery of nanoparticles into the tumor has recently resurfaced as the subject of investigation and discussion [167]. In experiments with liposomes labeled with anti-Her2-antibodies [168] and GNP labeled with transferrine [169], it was shown that functionalization improves the penetration of nanoparticles into cells; however, the contrast of particle accumulation in the tumor does not improved considerably. The biodistribution and localization of gold nanorods labeled with three types of probe molecules, including the (1) scFv-fragment of EGFR antibodies; the (2) N-terminal fragment of the peptide recognizing the urokinase plasminogen activator receptor (uPAR); and the (3) cycliс RGD-peptide recognizing the α _v_ β _3_ -integrin receptor have been studied [167]. It appears that all three types of ligands fail to significantly improve the contrast of particle accumulation in cell models and in the tumor upon intravenous administration, but they do have a considerable effect on extracellular distribution and intracellular localization. Therefore, a conclusion can be made that in the case of PPTT, the direct introduction of particles into the tumor can be more efficient than intravenous administration.

The last important question associated with modern PPTT has to do with the efficient delivery of radiation to the biotarget. Since the absorption of biotissue chromophores in the visible region is lower by two orders of magnitude than it is in the infrared region [138], the use of IR radiation dramatically reduces the nontarget thermal dose and increases the deep tissue penetration of the radiation. Nevertheless, the penetration depth typically does not exceed 5–10 mm [142, 170]; therefore, it is necessary to search for alternative solutions. The first approach consists in using impulse (nanoseconds) modes of radiation instead of continuous ones, which allow to increase the intensity of the irradiation without additional side effects. The second approach consists in using fibre-optic devices for endoscopic delivery of the radiation or delivery inside the tissue. The advantages and drawbacks of this approach are evident. Finally, radiation with deeper penetration, such as radio radiation [171], can be used for hyperthermia.

GNP conjugated with antibiotics and antibodies have also been used as photothermal agents to inflict selective damage to protozoa and bacteria [172, 173]. The data on some questions related to the use of PPTT can be found in books and reviews [139, 170, 174, 175]. The thorough review [138] warrants special attention.

**2.2. Photodynamic therapy using gold particles**

The photodynamic method [176] is applied in the therapy of oncological diseases, certain dermal or infectious diseases, and is based on the use of light-sensitive agents – photosensitizers (including dyes) and, typically, visible light of a certain wavelength. Most frequently, the sensitizers is introduced into the organism intravenously; it may also be administered applicatively or perorally. The agents for photodynamic therapy (PDT) can selectively accumulate in the tumor or other target tissues (cells). The affected tissues are radiated with laser light with a wavelength corresponding to the absorption maximum of the dye. In addition to the usual heat release due to absorption [6], the second mechanism is also significant. It is associated with the photochemical generation of singlet oxygen and the formation of highly active radicals inducing necrosis and apoptosis of tumor cells. PDT results in tumor malnutrition and death due to the damage inflicted on its microvessels. The major drawback of PDT is that the photosensitizers remain in the organism for a long period of time; as a result, the patient’s tissues remain highly sensitive to light. On the other hand, the use of dyes for the selective heating of tissues [6] is characterized by low efficacy due to the small absorption cross-section of chromophores.

It is well-known [177] that metal nanoparticles are efficient fluorescence quenching agents. However, it has been recently demonstrated [178] that the fluorescence intensity can be amplified by a plasmon particle, by locating molecules at optimum distance from the metal. Theoretically, this idea can be used to enhance the efficacy of PDT.

**Fig. 10:**
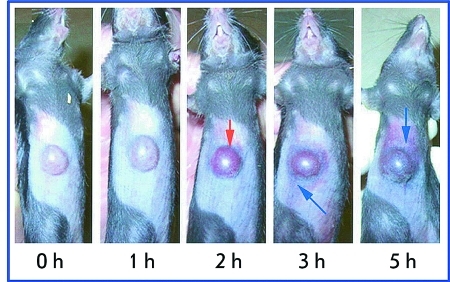
Accumulation of the GNP-TNF conjugates in mice tumors over 5 h after injection. A MC-38 tumor-burdened C57/BL6 mouse was intravenously injected with 15 µg of the GNP-TNF conjugates. The ventral surface of the animal was photographed at the indicated times, showing the color changes of the tumor over 5 hr. Red arrows show tumor uptake in the conjugates; blue arrows show accumulation of the conjugates in the tissues surrounding the tumor. Adapted from Ref. [191] by permission of The American Association for Cancer Research.

In a number of studies, the proposed method allowed to deliver drugs in polyelectrolyte capsules on GNP that disintegrate under laser radiation and deliver the therapeutic agent to the targets [179, 180] or to use nanoparticles surrounded by a layer of polymer nanogel [181, 182]. Moreover, photoactive agents [183] and peptides facilitating the intracellular penetration [184] are used within conjugates. It has recently been proposed [185] to use composite nanoparticles that, in addition to gold nanoshells, comprise magnetic particles, photodynamic dye, PEG, and antibodies. Finally, according to the data [186], nanoparticles conjugated with photodynamic dyes can have a synergetic antimicrobial effect.

Thus, gold nanostructures with plasmon resonance show promise for the selective PPTT of oncological and other diseases. However, it is clear that a number of questions require further study, such as: stability, biocompatibility, chemical interaction between nanoparticle conjugates in physiological environments, blood circulation time, penetration into the tumor, interaction with the immune system, excretion of nanoparticles, etc. We expect that the success in the initial stages in the use of nanoparticles for selective PPTT will be broadened to the clinical stage [138], provided that the optimal technical parameters are studied further.

**2.3. The use of gold nanoparticles as therapeutic agents**

Gold nanoparticles are increasingly actively being used not only in diagnostics and cell photothermolysis experiments, but also for therapeutic purposes. In 1997, the successful application of colloidal gold in a patient with rheumatoid arthritis was first reported [187]. In 2008, a vast array of data on the ten-year-long clinical trials of the preparation Aurasol ^®^ for peroral administration upon severe forms of rheumatoid arthritis was published [188]. The positive results achieved upon intra-articular introduction of colloidal gold into rats with collagen-induced arthritis were described [189]. The authors attribute the positive effect to an increase in anti-angiogenic activity due to the binding between GNP and the vascular endothelial growth factor and, therefore, the decrease in macrophage infiltration and inflammation. Similar results were obtained upon subcutaneous introduction of gold nanoparticles into rats with collagen- and pristan-induced arthritis [190].

Researchers from Maryland University used a colloidal gold vector to deliver the TNF to solid tumors in mice [191, 192]. Upon intravenous injection, GNP conjugated with TNF rapidly accumulates in tumor cells and is not detected in cells of the liver, spleen, and other healthy organs. Accumulation of GNP in the tumor is attested by the change in the color of the tumor; the tumor acquires a bright red/purple color (the color typical of colloidal gold and its aggregates), which coincides with the maximum of tumor-specific activity of the TNF ( *Fig. 10* ). The colloidal gold–TNF vector had lower toxicity and a higher efficacy in reducing tumor size in comparison with the native TNF, since maximum antitumor reaction was attained by using lower doses of the drug. The preparation for intravenous administration based on a GNP–TNF conjugate named AurImmune™ has already passed the second stage of clinical trials.

The antiangiogenic properties of GNP [193] were observed *in vitro* and *in vivo* . It turned out that GNP interact with heparin-binding glycoproteins – vascular permeability factors, growth factors of cardiac endothelium and fibroblasts. These agents mediate angiogenesis, including that in tumor tissues; therefore, GNPs inhibit their activity. Since intensive angiogenesis (the process of formation of new blood vessels in organs or tissues) is considered as one of the main tumor growth factors, the existence of antiangiogenic properties in GNPs could make them promising for tumor therapy. It was also demonstrated by the same researchers that gold nanoparticles enhance the apoptosis of the chronic lymphocytic leukemia cells that are stable to programmed death [194] and suppress the proliferation of multiple myeloma cells [195].

## 3. GOLD NANOPARTICLES AS DRUG CARRIERS 

The targeted delivery of drugs is one of the most promising and actively developing directions in the medicinal use of GNPs [196, 197]. Antitumor agents and antibiotics are the most popular objects of target delivery.

The options of using GNP conjugated with the following antitumor agents were proposed: paclitaxel [192], methotrexate [198], daunorubicine [199], hemcytabin [200], 6-mercaptopurine [201], dodecylcysteine [202], sulfonamide [203], 5-fluorouracil [204], platinum complexes [205], kahalalide [206], tamoxifen [207], herceptin [208], doxorubicin [209], prospidin [210], etc. The conjugation was carried out either by simple physical adsorption of the drugs onto GNPs or via the use of alkanethiol linkers. The effect of conjugates was assessed both (chiefly) on *in vitro* models, using tumor cell cultures, and *in vivo* , in mice with induced tumors of different natures and localizations (Lewis lung carcinoma, pancreatic adenocarcinoma, etc.). In addition to the active substance, target molecules (e.g., cetuximab) providing better anchoring and penetration of the complex into the target cells were used to design the delivery system. It was also proposed to use multimodal delivery systems, when a gold nanoparticle is loaded with several therapeutic agents (both hydrophilic and hydrophobic) and auxiliary agents, such as target molecules, dyes for photodynamic therapy, etc. [211]. Most researchers note high the efficacy of antitumor agents conjugated with gold nanoparticles.

Antibiotics and other antibacterial agents are also considered as objects that can be delivered by gold nanoparticles. The possibility of producing a stable complex of vancomycin and colloidal gold and the efficacy of such a complex against various enteropathogenic strains of *Escherichia coli* , *Enterococcus faecium* , *Enterococcus faecalis* (including vancomycin-resistant strains) have also been demonstrated [212]. Similar results were obtained in [213]: a complex of ciprofloxacin with gold nanoshells showed high antibacterial activity towards *E. coli* . The anti-leukemia drug 5-fluorouracil, conjugated with colloidal gold, has a noticeable antibacterial and antifungal effect against *Micrococcus luteus* , *Staphylococcus aureus* , *Pseudomonas aeruginosa* , *E. coli* , *Aspergillus fumigates* , and *A. niger* [214]. It should be noted that in all of the listed cases, the complexes of drugs with gold nanoparticles were stable, which could be attested by the optical spectra of conjugates.

On the contrary, stable complexes with gold nanoparticles could not be obtained for such antibiotics as ampicillin, streptomycin, kanamycin, hentamycin, neomycin, ciprofloxacin, gatifloxacin, and norfloxacin, which are active against *E. coli* , *M. luteus* , *S. aureus* , and *P. aeruginosa* [215–217]. Nevertheless, their activity when mixed with colloidal gold was higher by 12–40% than that of the antibiotic when used alone, depending on the antibiotic. On the basis of these data, the authors arrived at the conclusion that the antibacterial activity of antibiotics was enhanced by GNPs. However, the issue of the mechanisms underlying the possible boosting of the antibacterial effect of drugs has remained unsolved. It has been proved experimentally [218] that unbound gentamicin and a mixture of it with gold nanoparticles do not considerably differ in terms of their antimicrobial activity in tests on both dense and liquid nutrient media. It is speculated that stable conjugates of nanoparticles coated with antibiotic molecules are required to enhance the antibacterial activity. Thus, it was proposed to use the antibiotic cefaclor directly in the synthesis of GNPs. As a result, a stable conjugate was obtained. It was characterized by high antibacterial activity against *E. coli* and *S. aureus* .

There has been much less data on other drugs conjugated with gold nanoparticles. However, the high anti-oxidant activity of the tocoferol complex with gold nanoparticles should be noted, along with [220] the variants of its potential use that were proposed. Data has been published [22] indicating that, due to high local concentration, GNPs conjugated with the drug TAK-799 manifested a more pronounced activity against the human immunodeficiency virus, as compared with the drug itself. The procedure of per oral and intranasal introduction of insulin conjugated with colloidal gold was elaborated on rat models of diabetes mellitus. A decrease in blood sugar levels comparable with the effect of a subcutaneous introduction of insulin was reliably demonstrated [222]. Finally, the therapeutic effect of the antirheumatic drug etanercept conjugated with gold nanorods has been described [223].

In the end of this section, we would like to mention gene therapy, which seems to be the ideal strategy concerning genetics, as well as acquired, diseases [224]. Gene therapy implies an approach based on the introduction of genetic structures into cells and the organism for therapeutic purposes [225]. The desired effect was achieved either due to the expression of the inserted gene or by partial or complete suppression of the function of the damaged or overexpressed gene. Attempts to adjust the structure and function of the ill-functioning (affected) gene were recently made. In this case, gold nanoparticles can act as an efficient agent for delivering the genetic material into the cytoplasm and cell nucleus [226].

## 4. IMMUNOLOGIC PROPERTIES OF GOLD NANOPARTICLES 

Since the 1920s, researchers have shown keen interest in the immunological properties of colloidal metals (gold, in particular). This has been associated mainly with the physicochemical (non-specific) immunity theory proposed by J. Bordet, which postulates that immunogenicity and antigenic specificity depend predominately on the physicochemical properties of the compounds and, first and foremost, on their colloidal state. L.A. Zilber was successful in his attempts to obtain agglutinating sera from colloidal gold [227]. Moreover, it was shown in a number of studies that the introduction of a rigorous antigen, together with colloidal metals, stimulates the production of antibodies. Furthermore, it was found that certain haptenes adsorbed on colloidal particles can cause the formation of antibodies. In one of the best early reviews [228], a trove of data on the effect of colloidal gold on nonspecific immune reactions was provided [228]. In particular, it was noted that, 2 h after 5 ml of colloidal gold is introduced intravenously into rabbits, the leukocyte content in 1 ml of blood considerably increases (from 9900 to 19800) against a negligible decrease in mononuclear forms (from 5200 to 4900) and a considerable increase in polynuclear forms (from 4700 to 14900). It should be noted that such effects have not been observed upon the introduction of other colloidal metals. Unfortunately, with the development of immunology and the negation of many postulates in Bordet’s theory, interest towards the immunological properties of colloids has abated. However, the data on the amplification of the immune response to antigens adsorbed on colloidal particles has been used in the design of various adjuvants.

It is known that antibody synthesis is induced by agents that have an appreciably developed structure (immunogenicity). They include proteins, polysaccharides, and certain synthetic polymers. On the contrary, a considerable share of biologically active compounds (vitamins, hormones, antibiotics, narcotics, etc.) have a relatively low molecular weight and, therefore, cause a low immune response. In order to overcome this limitation in the standard methods used to produce antibodies *in vivo* , such agents (haptens) are chemically bound to high-molecular-weight carriers (most frequently, to proteins), making it possible to produce specific antisera. However, such antisera usually contain accompanying antibodies to the antigenic structures of the carrier [229].

In 1986, in a pioneering study by Japanese researchers [230], information on a successful attempt at producing antibodies to glutamic acid using colloidal gold particles as a carrier was published. A number of studies were subsequently published, in which this method was applied and developed in order to produce antibodies to the following haptens and rigorous antigens: amino acids, the platelet-activating factor, quinolinic acid, biotin, recombinant peptides, lysophosphatide acid, endostatin, peptides of viral capsid of B and C hepatitis, influenza, murrain, α-amidated peptides, actin, antibiotics, azobenzene, Аβ-peptide, clenbuterol, surface *Yersinia* antigens, transmissible gastroenteritis virus, and tuberculin (see review [231] and refs. therein). In all of the works listed, hapten was directly conjugated with colloidal gold particles and mixed with Freund’s complete adjuvant to immunize animals. As a result, sera with a high titre were obtained. The sera required no further purification to remove ballast antibodies.

In 1993, it was suggested that hapten (gamma-aminobutyric acid) be bound to the carrier protein before its conjugation with colloidal gold [232]. The proposition was supported in the studies devoted to the production of antibodies to a number of peptides, amino acids, phenyl-β-D-thioglucoronide, and diminazene (see [231 and refs. therein). The antibodies obtained through this procedure were characterized by both a high specificity to antigens and a higher titre (“extremely high, according to [232]”) –from 1 : 250000 to 1 : 1000000, in comparison with those produced using a routine method. The ImmunoSolution company currently offers antibodies to a number of neurotransmitters and amino acids. These antibodies are produced according to the procedure in [232].

In 1996, the possibility of using colloidal gold particles in the antiviral vaccine as the carriers of protein antigen of the capsid of the tick-borne encephalitis virus was first demonstrated [233]. Despite the fact that the vaccine contained no adjuvants, the experimental vaccine had better protective properties as compared with its commercial analogues.

A significant number of studies devoted to the use of GNP in designing DNA vaccines with gene constructions encoding proteins, to which antibodies had to be produced, have been published. In the case of efficient gene expression, these proteins serve as antigens for the development of the immune response. Colloidal gold particles are the most popular examples of nanoparticles–DNA carriers [234].

The technology used to produce antibodies against various antigens using colloidal gold as a carrier and adjuvant was described in [233, 235]. In this case, antigens are adsorbed directly at the surface of gold nanoparticles without using any binding agents. It was ascertained that the immunization of animals with an antigen conjugated with colloidal gold (both using Freund’s complete adjuvant and without it) results in the obtainment of specific antibodies with a high titre to a wide range of antigens without ballast antibodies. Gold nanoparticles can stimulate antibody synthesis in rabbits, rats, and mice if a lower dose of the antigen is used in comparison with the amount that is required when using a number of conventional adjuvants ( *Table 2* ).

**Table 2:** Indices of antibody titres during immunization of rabbits with yersiniose antigen (according to [235])

Preparation	First immunization	Second immunization	Reimmunization
Colloidal gold + antigen (1 mg)	1:32	1:256	1:10240
Freund’s complete adjuvant + antigen (100 mg)	1:32	1:256	1:10240
Physiological solution + antigen (100 mg)	1:32	1:256	1:512

Gold nanoparticles used as antigen carriers were shown to stimulate the phagocytic activity of macrophages and affect the functioning of lymphocytes, which probably is responsible for their immune-modulating effect. Moreover, gold nanoparticles and their conjugates with low- and high-molecular-weight antigens stimulate the respiratory activity of the cells of the reticulo-endothelial system and the activity of the mitochondrial enzymes of macrophages [236], which may be one of the causal factors behind the adjuvant properties of colloidal gold. The fact that gold nanoparticles act both as a carrier and an adjuvant (i.e., represent haptens to T-cells) should be considered as the most interesting side of the manifestation of the immune properties of colloidal gold. In particular, gold nanoparticles conjugated with antigens affect T-cell activation: a tenfold increase in the proliferation, as opposed to that upon the addition of a native antigen, was detected. This provides evidence in support of the fact that it is fundamentally possible to act directly on T-cells with the subsequent activation of macrophages and destruction of a pathogen.

However, none of the studies contains data on the mechanisms that underline these properties of gold particles. We consider the discussion in [232] on the preferable macrophage response to corpuscular antigens, as opposed to the soluble ones, to be undoubtedly reasonable. The researchers who study the mechanisms of action of DNA vaccines and use gold nanoparticles to deliver genetic material into the cell also confirm this fact [235]. The role of Kupfer cells and Langerhans cells in the formation of the immune response was revealed in these studies. The effect of dendrite cells on the formation of the immune response upon the introduction of an antigen conjugated with gold nanoparticles was discussed in [237]. Moreover, it was noted that when using nanoparticles in medical practice, one should make sure that there are no lipopolysaccharides on their surface. The recent studies [238, 239] were devoted to the interaction between the cells of the immune system and gold nanoparticles.

The penetration of peptide-conjugated GNP into macrophage cytoplasm resulting in their activation was shown by electron microscopy [240]. It was ascertained that after the conjugates interact with the TLR-4 receptors of macrophages, the nanoparticles penetrate into the cell, which is accompanied by the secretion of inflammatory cytokines – TNF, interleukin-1β and interleukin-6–and the inhibition of macrophage proliferation. Upon the introduction of GNP, the amount of macrophages decreases, while their size increases [241]. The level of interleukin-1 and interleukin-6 and TNF also increases. Another (noninflammatory) mechanism of penetration of gold nanoparticles into macrophages – by interaction with scavenger receptors---is not improbable [242]. The effect on nonconjugated colloidal gold on immune-competent cells *in vivo* was studied [243]. It was shown that the introduction of GNP into mice results in an increase in the proliferation of lymphocytes and normal killers and an increase in interleukin-2 production.

We believe that the detection of adjuvant properties in GNP creates favorable conditions for the development of a new generation of vaccines. 

## 5. BIODISTRIBUTION AND TOXICITY OF GOLD NANOPARTICLES 

All the facts mentioned above are proof that GNP have recently been actively used in different spheres of nanomedicine for diagnostic and therapeutic purposes. Moreover, they are being introduced parenterally into the organism of animals and humans with increasing frequency. The acute questions concerning their biodistribution, blood stream circulation, pharmacokinetics and removal from the organism, as well as possible toxicity at the level of the entire organism or at the level of cyto- and genotoxicity, emerged almost at the same time when GNP started to be used in medicine. It should be noted that data on the biodistribution and toxicity of GNP at the time of writing remained scarce and inconsistent. 

It was demonstrated by the analysis of published data that the burst in activity regarding investigations into the biodistribution and toxicity of GNP took place during the past 3–4 years [7, 244–248]. Since numerous research groups started their projects independently, there is a vast dispersion in the experimental design, including the size and shape of particles, functionalization methods, types of animals, doses and methods of particle introduction, etc. As a result, there has been serious discrepancies in the data and conclusions on the level and kinetics of biodistribution for toxicity estimations, as well. Yet, certain tentative conclusions can still be made.

Firstly, the organs of the reticuloendothelial system serve as the main target for the accumulation of 10–100 nm GNP; biodistribution homogeneity decreasing with decreasing size. The rapid reduction in particle concentration in blood and their prolonged retention in the organism is associated with the functioning of the hepatobiliary system. Since it takes 3 to 4 months for the accumulated particles to be excreted from the liver and spleen, the question of the doses and possible inflammatory processes is of paramount importance. 

Secondly, the available data allows for the reasonable assumption that the effect of nanoparticle penetration via the hematoencephalic barrier depends critically on their size; 5–20 nm being the upper limit. Thirdly, gold nanoparticles 1–2 nm in diameter could be more toxic due to the possibility of irreversible binding to the biopolymers in cells. Also, numerous experiments on cell cultures have revealed no observable toxicity in colloidal particles with a size of 3–100 nm, provided that the threshold dose does not exceed a value of the order of 10 ^12^  particles/ml.

Data on *in vivo* experiments is scarce and somewhat inconsistent. It can only be assumed that there is no observable toxicity upon the short-term (approximately one week long) introduction of GNP at a daily dose lower than 0.5 mg/kg.

**Fig. 11:**
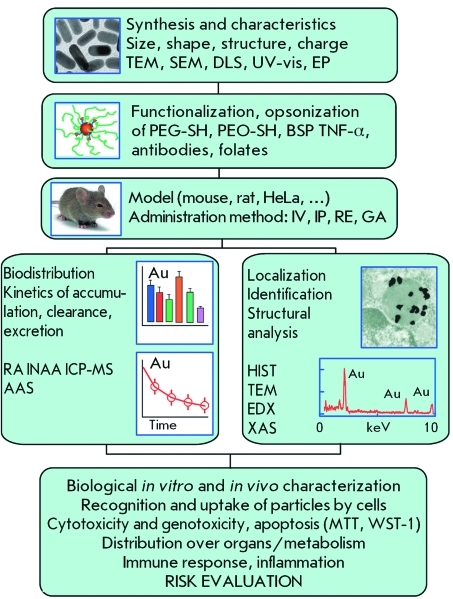
Scheme of biodistribution and nanotoxicology experiments. The first step is the fabrication of desired particles and the characterization of their size, shape, structure, charge by transmission or scanning electron microscopy (TEM, SEM), dynamic light scattering (DLS), UV-vis spectroscopy at the ensemble (suspension) and single-particle levels, electrophoresis, and other methods. The second step includes the functionalization of the particle surface with appropriate ligands, including thiolated PEG or poly(ethylene oxide) (PEO) molecules, tumor necrosis factor (TNF-a), antibodies, and folates, as well as opsonization with blood serum proteins (BSP, like albumin etc.). Conjugates are administered to models in accordance with the experimental design, i.e., by using selected doses and routes of exposure, including intravenous (IV), intraperitoneal (IP), respiratory (RE), or gastrointestinal (GA). The biodistribution into organs and the kinetics of accumulation/clearance are determined according to a selected time-dependent scheme of tissue sampling. Samples are analyzed by radioactive analysis (RA), instrumental neutron activation analysis (INAA), inductively coupled plasma-mass spectrometry (ICP-MS), and atomic absorption spectroscopy (AAS). Particles and related structures also can be identified at the tissue (histological, HIST) and cellular levels by electron microscopy (SEM, TEM), energy dispersive X-ray spectroscopy (EDX), and X-ray absorption spectroscopy (XAS). The final step is the integration of data for the biological characterization of GNP effects and the evaluation of possible risks through the use of cellular-level information (cellular recognition and penetration, cytotoxicity, genotoxicity, and apoptosis/necrosis; MTT and WST-1 assays) and at the whole organism level (organ distribution, accumulation and clearance/excretion, degradation and metabolism, immunogenicity, and inflammation). Reproduced from Ref. [248] (http://dx.doi.org/10.1039/c0cs00018c) by permission from The Royal Society of Chemistry.

Recent data underscore the interest generated by and intensity of studies in the sphere of nanotoxicology, whose number has exploded. *Fig. 11* shows the general scheme of a study of the biodistribution and toxicity of nanoparticles, which can be used for planning experiments [248]. For a better insight into the problems of the biodistribution and toxicity of GNP, we recommend reviews [244–248].

## CONCLUSIONS 

Thanks to the rapid development in technologies for the chemical synthesis of GNP over the past decade, a great variety of particles with different sizes, shapes, structures, and optical properties are now available to contemporary researchers. Moreover, the question of the simulation of nanoparticles that would possess the desired physical (optical, thermal, etc.) properties, with subsequent development of the procedures for synthesizing the simulated structures, is now on the agenda. 

In terms of applications in medicine, the development of efficient technologies for the functionalization of GNP with different classes of molecules providing stabilization *in vivo* and directed interaction with biological targets is of significance. Today, thiolated derivatives of PEG and other molecules are considered to be the best stabilizing agents. In particular, PEG-coated particles can remain in the blood flow for a longer time and are less susceptible to attacks from the cell components of the immune system.

It is now widely accepted that GNP conjugates are excellent labels for solving the problems of bioimaging, which can be implemented using various optical technologies, including resonance scattering dark-field microscopy, confocal laser microscopy, different variants of two-photon luminescence of GNP, optical coherence tomography, acoustic tomography, etc. 

GNP conjugates have found application in analytic studies that can be based both on modern instrumental methods (surface-enhanced Raman spectroscopy, LISNA, IR Fourier spectroscopy, etc.) and on the use of simple solid-phase or homophase procedures (dot analysis, immunochromatography). Two examples can be given as illustrations: (1) the prostate-specific antigen can be determined using GNP conjugated with antibodies with a sensitivity that is higher than that in the conventional immunoenzyme assay by a factor of 1,000,000 [249]; (2) the strict dependence of color on interparticle distances allows visual detection of mutant DNAs in the so-called “Northwestern spot test” [68]. Along with the examples of clinical diagnostics of cancer, Alzheimer’s disease, HIV, hepatitis, tuberculosis, diabetes mellitus, and other diseases, new diagnostic applications for GNP should be expected.

Plasmon photothermal laser therapy of cancer using GNP was first described in 2003 and recently moved into the stage of clinical approval. The actual clinical success of this technology will depend on how quickly several urgent problems can be solved: (1) developing efficient methods for the delivery of radiation to tumors inside the organism using fibre-optic technologies or nonoptical heating methods; (2) elaborating methods for delivering conjugates to tumors, enhancing the contrast and uniformity of accumulation; and (3) developing methods for controlling the *in situ* photothermolysis process.

Targeted delivery of DNA, antigens, and drugs using GNP is one of the most promising directions in biomedicine. In particular, the studies performed by Jiang *et al* . of the University of Toronto [250] have revealed the size-dependent possibility of herceptin conjugated GNP into tumor cells with a much higher efficacy in comparison with that of the pure preparation. The recent critical revision of the PPTT concept based on the intravenous-targeted delivery of GNP conjugated with molecular probes to tumor receptors [167] points to the necessity to continue studies in this direction. In view of the data in [167], it seems quite reasonable to use the “non-targeted” PEG-coated gold nanoshells of the SiO _2_ /Au type with a nucleus diameter of 120 nm and thickness of the gold layer of 15–20 nm as a universal marker for PPTT and bioimaging [25, 41]. It should be emphasized that N. Halas, J. West, *et al* . of Rice University (United States) started clinical trials of these particles for PPTT in 2010.

Finally, there is a necessity to continue and broaden studies of the biodistribution and the toxicity of GNP. First of all, a coordinated program is required, which would reveal the correlations between particle parameters (size, shape, functionalization with various molecular probes), experimental parameters (model, doses, method, and administration scheme, observation duration; organs, cells, subcellular structures under study, etc.), and the observed biological effects. Coordinated efforts in the introduction of standards for the particles and methods used for the testing of nanomaterial toxicity are also required. 
